# Evaluating the Efficacy and Risks of Early Lecanemab Treatment in APOE4 Humanized Alzheimer's Disease Mouse Models

**DOI:** 10.1002/alz70855_102371

**Published:** 2025-12-27

**Authors:** Arash Salahinejad, Amr Eed, Mohammad H Alipour, Kellly Summers, Kate M. Onuska, Luke Smolders, Ebrima Gibbs, Czarina Evangelista, Lisa M Saksida, Timothy J Bussey, Neil R Cashman, Johanne Kaplan, Scott Napper, Taylor Schmitz, Vania F Prado, Ravi S. Menon, Marco AM Prado

**Affiliations:** ^1^ University of Western Ontario, London, ON, Canada; ^2^ Robarts Research Institute, London, ON, Canada; ^3^ Schulich School of Medicine and Dentistry, London, ON, Canada; ^4^ University of British Columbia, Vancouver, BC, Canada; ^5^ ProMIS Neurosciences, Cambridge, MA, USA; ^6^ University of Saskatchewan, VIDO, Saskatoon, SK, Canada; ^7^ Lawson Health Research Institute, London, ON, Canada; ^8^ Centre for Functional and Metabolic Mapping, Robarts Research Institute, London, ON, Canada

## Abstract

**Background:**

Alzheimer's disease (AD) is the leading cause of dementia. AD disproportionately affects APOE4 carriers, who experience accelerated amyloid β (Aβ) accumulation, including cerebral amyloid angiopathy (CAA). While monoclonal antibodies (mAbs) like lecanemab reduce amyloid plaques, their use is complicated by amyloid‐related imaging abnormalities (ARIA‐likely related to brain bleeds), particularly in APOE4 carriers. Lecanemab, a humanized mAb targeting Aβ protofibrils, but still poses an enhanced risk for APOE4 carriers. This study explores whether early lecanemab treatment can reduce the risk of microbleeds and prevent cognitive deficits as well as brain atrophy in APOE4 humanized AD mouse models.

**Methods:**

Advanced AD mouse models with humanized APP (hApp), Tau (hMAPT), and APOE3/APOE4 genes were used. The murine version of lecanemab (mAb158) was synthesized and shown to target synthetic Aβ oligomers and pre‐fibrillar amyloid in these models. Starting at 3 months of age, hApp^NL‐F^‐hMAPT‐APOE4 and hApp^NL‐F^‐hMAPT‐APOE3 mice received mAb158 (20 mg/kg weekly for 26 weeks) or vehicle (PBS). Cognitive performance was assessed at 6, 9, and 12 months using a touchscreen‐based Continuous Performance Test (CPT) to assess attention. At study end, brain tissues were analyzed via light‐sheet microscopy, immunohistochemistry, Prussian Blue staining, and ELISA to evaluate plaques, amyloid deposits, microbleeds, and Aβ levels.

**Results:**

Immunohistochemistry and light‐sheet microscopy revealed significant amyloid accumulation in the brain and blood vessels of hApp^NL‐F^‐hMAPT‐APOE4 mice, from 6 months of age onward, compared to hApp^NL‐F^‐hMAPT‐APOE3. In the mAb158 treated mice, insoluble Aβ accumulation was negligible in both genotypes at 9 months of age and significantly reduced at 15 months of age in hApp^NL‐F^‐hMAPT‐APOE4 mice compared to vehicle. hApp^NL‐F^‐hMAPT‐APOE4 mice showed CPT deficits starting at 6 months under vehicle and mAb158, with no difference in performance between treatment groups. Preliminary analyses suggest mAb158 causes microhemorrhages.

**Conclusions:**

Early mAb158 treatment reduces key Alzheimer's disease pathology but fails to prevent attention deficits in hApp^NL‐F^‐hMAPT‐APOE4 mice and may increase microbleeds in this vulnerable group. By combining new generation mouse models of genetic AD risk with translational cognitive and imaging biomarkers, we propose a preclinical platform to better predict the safety and efficacy of monoclonal antibody treatments.

